# An audit of dementia education and training in UK health and social care: a comparison with national benchmark standards

**DOI:** 10.1186/s12913-019-4510-6

**Published:** 2019-10-21

**Authors:** S. J. Smith, S. Parveen, C. Sass, M. Drury, J. R. Oyebode, C. A. Surr

**Affiliations:** 1 Centre for Dementia Research, School of Health and Community Studies, Leeds Beckett University, Leeds, LS1 3HE UK; 2 Centre for Applied Dementia Studies, Faculty of Health Studies, University of Bradford, Bradford, BD5 0BB UK

**Keywords:** Dementia, Education, Training, Audit, Workforce development

## Abstract

**Background:**

Despite people living with dementia representing a significant proportion of health and social care users, until recently in the United Kingdom (UK) there were no prescribed standards for dementia education and training. This audit sought to review the extent and nature of dementia education and training offered to health and social care staff in the UK against the standards described in the 2015 Dementia Training Standards Framework, which describes the knowledge and skills required of the UK dementia workforce.

**Methods:**

This audit presents national data concerning the design, delivery, target audience, length, level, content, format of training, number of staff trained and frequency of delivery within existing dementia training programmes offered to health and social care staff. The Dementia Training Standards Framework was used as a reference for respondents to describe the subjects and learning outcomes associated with their training.

**Results:**

The findings are presented from 614 respondents offering 386 training packages, which indicated variations in the extent and quality of training. Many training packages addressed the subjects of ‘*person-centred care’, ‘communication’*, ‘*interaction and behaviour in dementia care’*, and ‘d*ementia awareness’*. Few training packages addressed subjects concerning ‘*pharmacological interventions in dementia care’*, ‘*leadership’* and ‘e*nd of life care’*. Fewer than 40% of The Dementia Training Standards Framework learning outcomes targeted to staff with regular contact with people with dementia or in leadership roles were covered by the reported packages. However, for training targeted at increasing dementia awareness more than 70% of the learning outcomes identified in The Dementia Training Standards Framework were addressed. Many training packages are not of sufficient duration to derive impact; although the majority employed delivery methods likely to be effective.

**Conclusions:**

The development of new and existing training and education should take account of subjects that are currently underrepresented and ensure that training reflects the Training Standard Framework and evidence regarding best practice for delivery. Lessons regarding the limitations of training in the UK serve as a useful illustration of the challenge of implementing national dementia training standards; particularly for countries who are developing or have recently implemented national dementia strategies.

**Electronic supplementary material:**

The online version of this article (10.1186/s12913-019-4510-6) contains supplementary material, which is available to authorized users.

## Introduction

There are approximately 50 million people living with dementia worldwide [1], a number set to double every 20 years making dementia an international health priority [2]. Health and social care is a complex system that operates on many levels across different organisations. The health and social care workforce must be equipped with appropriate knowledge and skills to support the needs of people with dementia. This workforce includes any person who may come into contact with people living with dementia, or suspected dementia, in health and social care settings from the point of diagnosis to end of life. This may include healthcare professionals, allied healthcare professionals, pharmacists, general practitioners, clinical and non-clinical staff, porters, kitchen staff, and receptionists. People living with dementia represent a significant proportion of health and social care service users; up to 40% of patients in hospital [3] and 80% of people in residential care [4] live with dementia. Supporting people with dementia requires a range of skills since people living with dementia can experience behavioural changes influenced by cognitive impairment as well variations in general health and social circumstances [5]. Person-centred care has been widely adopted as a values-based approach to supporting people with dementia [6]. For this reason, person-centred dementia care is mandated in national [7] and international guidance [2]. High quality education and training are essential for delivering high quality dementia care, and ensuring appropriate caregiving strategies are used [8].

Despite the importance of education and training for the workforce, in the UK there is currently no mandated requirement for accredited training. Consequently, poor levels of knowledge about dementia in the workforce are common [9]. In response to the rising prevalence of dementia and associated pressure on health and social care services [3] the UK government has set targets to increase the number of staff in receipt of dementia training [10, 11]. A national framework setting out the expectations regarding educational content and learning outcomes for England was published in October 2015 [12]. The Dementia Training Standards Framework, hereafter The Framework [13], outlines the essential subjects and learning outcomes that health and social care staff should accomplish to deliver an acceptable standard of dementia care. The Framework is divided into three tiers of Learning Outcomes (LOs). Tier 1 refers to training that all health and social care staff (as described above) should receive; LOs within this tier of training promote dementia awareness. For example porters, catering or kitchen staff should receive this training. Tier 2 sets out basic skills and knowledge relevant to any staff who have regular contact or provide direct care for people with dementia, such as registered nurses or care support staff. Tier 3 sets out advanced knowledge for leaders in the field of dementia care such as health and social care mangers, ward managers, dementia champions or dementia care trainers.

Whilst providing guidance for content, The Framework does not take account of the pedagogical considerations of training. The diversity of the dementia workforce presents unique challenges for providers. The majority of the workforce comprises unqualified care staff who are low paid, have low levels of literacy and numeracy, and who may have English as a second or additional language [14, 15]. Qualified staff in the workforce typically have degree level education, but accredited training generally lacks substantive dementia content [14]. A recent review sought to establish the common features of high quality educational provision for the dementia care workforce [16]. The review adopted Kirkpatrick’s [17] model to conceptualise impact of training, whereby training with greater impact influences behaviour as well as knowledge. The findings indicated that education and training programmes with greatest impact were tailored to the recipient staff groups and relevant to their role and experience i.e. avoiding a one fits all approach. Better education and training included active participation (such as group discussions and activities) that underpinned practice-based learning with theory. Effective education and training lasted at least eight hours in total, with sessions of 90 min or more, and was delivered by an experienced facilitator. It also provided opportunities to support the application of learning in practice.

Although there have been policy initiatives designed to increase dementia education and training, no registration or accreditation for dementia training exist therefore there is no assurance regarding training quality. Having established The Framework for the ideal content of dementia training and identified effective approaches toward the delivery of education and training, it is now possible to better understand and describe education and training that is likely to be effective. The audit presented in this paper was funded by the National Institute for Health Research Policy Research Programme on behalf of Health Education England (HEE) within the *What Works* study with the aim of ascertaining the extent, nature and quality of education and training in dementia in the UK. The audit findings will thus enable recommendations regarding areas for development of education and training to better equip the workforce.

## Methods

### Audit design

Each respondent to the audit was required to report the number and nature of training packages they provided. For each training package reported the delivery method/format of the training, target audience, length, content and level of training, number of staff trained and frequency of delivery was audited. In terms of content, the subject(s) and learning outcomes covered in the training or education (with reference to the Framework) provided by or within the organization was audited. Responses included open and closed force choice questions (see Additional file 1 for audit^1^). The audit was piloted with members of the project stakeholder monitoring group which included representation from the care home sector, primary care practitioners, hospital dementia leads, commissioners of training, and lay advisors with experience of supporting people with dementia.

### Setting and distribution

Using an online web-based survey tool (Snap surveys; see https://www.snapsurveys.com/) an audit was distributed in England using databases of health and social care providers, training providers and training commissioners. We included commissioners as in the UK budgets and strategies for training provision for the health and social care workforce can sit with local commissioners of health and social care services, for example clinical commissioning groups or local authorities.

The distribution databases included extant resources held at the research sponsor University, national databases of professional networks (such as HEE distribution lists) and online lists of health and social care providers i.e. NHS Trust Research and Development (R&D) offices, care home groups (www.carehome.co.uk accessed Nov 2015). In total 1621 invitation emails were sent from the University databases, the contacts were primarily from health and care organisations in the local region consisting private sectors and NHS (primary care practice, hospitals, trusts and clinical commissioning groups). The researchers also contacted 90 community pharmacies and local authorities. A further 226 individual care homes and care home groups were contacted from the directory held at carehome.co.uk. The direct emails were predominantly directed to lead nurse practitioners, directors or managers (clinical, finance, HR), education or training leads. Where participation was targeted, organizations were sent an email with an overview of the purpose of the research including a link to the online audit. Completion reminders included timed follow up e-mails. Each recipient received two reminders to complete the audit at two week intervals, after which no follow up was made.

The audit was also advertised via articles and adverts in journals read by the staff population and the use of social media including Facebook and Twitter. The promotion of the audit included a direct link to the survey.

### Audit analysis

The data was downloaded as one export from Snap Surveys into Microsoft Excel one month after the last reminders for completion were sent. Data cleansing included removing incomplete audit responses as described in the results section. Data was subsequently interrogated at the respondent and training package level. At the package level the degree to which training covered the subjects and learning outcomes identified in The Framework was analysed.

## Results

### Overview of respondents

In total 614 respondents commenced the audit. Of these 178 did not provide essential information and were excluded from analysis. Of the 436 remaining respondents (234 Care Providers, 129 Training providers and 47 Commissioners), 195 did not provide information about their training and were subsequently excluded. There were 241 respondents who provided data pertaining to at least one package (386 packages reported in total); an overview is provided in Table 1.

**Table 1:** Respondents, organisation type and number of education or training packages provided

Provider Type	Organisation Type	Number Respondents	Number of Packages
Care Provider	Hospital Care	44	64
	Residential Care	32	45
	Community Mental Health Trust, Mental Health Trust or Community Pharmacy	23	31
	Charitable Care	16	28
	Other Care Provider (E.g. Extra Care)	14	19
	Primary Care	9	12
	Domiciliary care	5	9
	TOTAL CARE PROVIDER	143	208
Training Provider	University	40	74
	Other Training Provider	18	30
	Private Training Company	17	39
	Charitable Organisation	12	20
	TOTAL TRAINING PROVIDER	87	163
Commissioning Group/Network	COMMISSIONERS TOTAL	11	15
	TOTAL	241	386

The majority of respondents were from care provider settings; hospital and residential care reported the highest number of packages. The fewest responses were received from domiciliary and primary care providers. In the training provider sector, most respondents came Universities. Overall fewest responses came from commissioners, although training provided from this sector reached large numbers of learners.

### Delivery methods

Respondents provided detail regarding the delivery methods used for each package. Across the 386 packages the most commonly used method was face-to-face training or education in small groups (see Table 2). Few respondents reported using one-to-one approaches. Use of e-learning methods were reported by only 13% of respondents, although some respondents included online interactive tutorials under Other category, so this may be an underestimated. The Other category included use of online interactive tutorials, film and music, workshops and simulation. Respondents were able to assign more than one method to each package; some methods were used as stand-alone approaches and some concurrently.

**Table 2:** Methods of delivery and recipients for 386 training packages reported

Method of Delivery	N (%) packages	Recipients of Training	N (%) packages
Face to Face Small (less than 20)	202 (52%)	Unqualified Clinical Staff	117 (30%)
Face to face large (more than 20)	81 (21%)	Qualified Clinical Staff	102 (26%)
E learning	51 (13%)	Ancillary Staff	29 (8%)
One to One	22 (6%)	Unit/Ward managers	66 (17%)
Practice Based Learning	62 (16%)	Service managers	57 (15%)
Other	20 (5%)	All Staff	189 (49%)
		Other	48 (12%)

### Recipients of training

Detail concerning the recipients of training are reported in Table 2. Across the 386 packages around half of the packages were targeted to all staff groups, whilst 30% were delivered to unqualified clinical or care staff (e.g. nursing assistant, support worker, residential care worker) and around 26% to qualified clinical or care staff (e.g. general practitioner, psychologist, nurse). Fewest packages were delivered to ancillary and clerical staff (e.g. porter, secretary, domestic staff). Respondents were able to identify that the training targeted more than one group. Respondents indicated that 12% of packages were delivered to “other” staff not listed in the audit options; these included people working with dementia in roles where they deliver training, volunteers and family members, members of public and schools.

### Length of training

The audit captured how many hours’ packages focused on dementia. Duration of the training was reported in hours for 299 out of 386 packages. The median length of the training was six hours (range 1–1800 h). Of the 299 packages, 102 (34%) packages lasted more than 8 h; previous research suggests training should be a minimum of eight hours [16]. Length of training in days was reported for 236 packages. The median length of the training was one day (range 1–1095), 48% of packages (114) lasted for more than one day. The training with the longest duration represented a three year part-time MSc programme.

Details of how many times each package had been delivered were reported for 216 packages (median 8 times, range 1–300). Two hundred and thirty-nine packages reported the number of learners for each package (median 107 learners, range 7–15,000), and 60 of the packages had been delivered to more than 500 learners. Higher numbers of learners were related to the package being delivered multiple times or to multiple organisations. One NHS trust delivered two programmes 100–150 times to 8000 learners in total. Overall 93 packages were delivered to multiple organisations. One training company delivered their package to 460 organisations and 15,000 learners across all types of health services, a fire service and shopping centres.

Respondents indicated that 352 of the packages covered at least one of the subjects identified in The Framework. The subjects that were most commonly addressed were ‘*person-centred care’*, ‘*communication’, ‘interaction & behaviour in dementia care’*, and ‘d*ementia awareness’* (see Table 3); over 300 packages addressed these subjects. Significantly fewer packages addressed ‘*pharmacological interventions in dementia care’*, *‘leadership’* and ‘*end of life care’*.

**Table 3:** Overview of Packages; Subjects and Learning Outcomes (LOs)

	No. of packages that address subject	No. of packages that addressed at least one subject level LO	Diff. between predicted and actual coverage of LOs	Number of LOs in Subject	Av. Number of LOs addressed	% of LOs addressed
Dementia Awareness	317	306	11	11	8.91	81
Dementia Identification, Assessment & Diagnosis	201	197	4	19	10.84	57
Dementia Risk Reduction & Prevention	182	174	8	10	5.03	50
Person-centred Dementia Care	332	289	43	11	7.85	71
Communication, Interaction & Behaviour	322	285	37	18	13.91	77
Health & Wellbeing in Dementia Care	270	238	32	18	10.04	56
Pharmacological Interventions in Dementia Care	112	103	9	14	6.63	47
Living Well with Dementia & Promoting Independence	298	249	49	17	8.97	53
Families and Carers as Partners in Dementia Care	267	227	40	18	11.19	62
Equality Diversity & Inclusion in Dementia Care	241	197	44	13	6.05	47
Law, Ethics & Safeguarding	168	140	28	16	9.08	57
End of Life Dementia Care	139	108	31	11	5.29	48
Research & Evidence Based Practice in Dementia Care	176	96	80	9	2.09	23
Leadership in Transforming Dementia Care	115	109	6	10	6.86	89

For respondents who indicated that their package addressed a subject, the audit went on to require them to identify which LOs associated with the subject had been addressed by the training. A number of respondents indicated that they addressed a subject, but were unable to identify any LOs that the training addressed i.e. they overestimated the degree to which their training addressed the subject as described by The Framework (see Table 3). This was most apparent for the subject ‘*research and evidence based practice’*; 176 respondents believed their packages addressed this subject but only 96 packages reported addressing at least one LO described in The Framework. Conversely, all but four respondents who identified addressing the subject ‘*assessment and diagnosis of dementia’* addressed at least one LO from The framework.

In assessing the degree to which the content of the training met the needs of the dementia workforce, the proportion of LOs addressed for each subject area was interrogated. The mean number of LOs covered in each subject area was calculated. Packages focused on ‘*leadership in transforming dementia’ care* covered 89% of the LOs described in the Framework- suggesting that although there were fewer packages addressing this subject, those that did provided education and training in accordance with The Framework. Similarly, packages addressing ‘*dementia awareness’* delivered training largely aligned to the framework (81% of LOs covered). However, packages focused on ‘*research & evidence based practice in dementia care’* only delivered 23% of the LOs described in The Framework. For ‘*pharmacological interventions in dementia care’*, ‘*equality diversity & inclusion in dementia care’* and ‘*end of life dementia care’* fewer than 50% of the LOs from The Framework were addressed.

Of the 386 packages, 137 (35%) had an assessed component, whilst only 98 (25%) were accredited. There were a range of accreditation types, from academic accreditation to vocational accreditation e.g. National Vocational Qualification (NVQ), and Continuing Professional Development (CPD). Some reported endorsement from the third sector such as the Alzheimer’s Society.

**Fig. 1:**
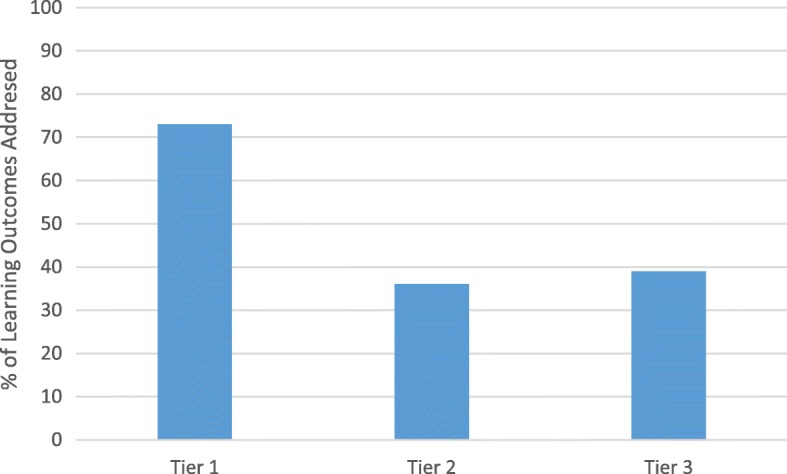
Percentage of Learning Outcomes addressed at each tier of training

The final consideration of the audit was establishing the level at which training was delivered. Of the 352 education and training packages delivered the mean proportion of LOs addressed was established from the total number of LOs described in each tier of the framework (Tier 1 = 73%, Tier 2 = 36%, Tier 3 = 39%), see Fig. 1. These findings indicated that whilst Tier 1 packages address over 70% of the LOs described in The Framework, less than 40% of the LOs were covered at Tier 2 and Tier 3.

## Discussion

The findings of this audit highlight the variability in the degree and nature of education and training in dementia care across the UK. The national dementia strategy in the UK has been in place for 14 years; but only relatively recently (2015) has a national standard of dementia of education and training been described. Internationally, countries have adopted a variety of approaches to improve the quality of life of people with dementia; some have launched policies, others plans, strategies or frameworks [1]. There is considerable variability with regards to how much these plans reflect a need to improve dementia related education and training. These audit findings will have relevance for countries wishing to improve or develop education and training. For countries seeking to develop standards of education and training the findings suggest avoiding a one fits all approach; ensuring that a breadth of training at all levels is available to staff. For example in Malaysia where a dementia strategy has only recently been adopted which is predicated in the UK model [18].

The responses in the current audit were obtained from a range of health and social care settings in the UK. Many respondents came from hospital settings and care homes providers, where dementia care is prioritised as many service users live with dementia [4], and there are significant costs associated with inadequate care [3]. There were high numbers of respondents from University organisations which reflected a range of approaches to programme delivery ranging from dedicated dementia specific programmes (e.g. postgraduate dementia courses) to dementia training that is integrated with professional training (e.g. dementia specific mentorship on placement). This may reflect an increased awareness of the importance of dementia in interprofessional training [19]. In contrast, lower numbers of responses from primary care may be because these organisations are generally small-scale and professional training is often organised on an individual basis rather than by the provider, who may not be aware of the extent and nature of training staff have received.

The audit considered the pedagogy of the education and training provided; an important consideration given that there is established universal evidence base regarding good pedagogical practice. For example, it is well established that assessment is an important component of learning [20]. Only 35% of packages indicated that they used assessment. One of the limitations of the current study was that we did not ask participants whether learning outcomes had been defined at the outset of the training. From a pedagogical perspective establishing learning outcomes at the outset of training is critical to ensure that the educator and learner have a clear understanding of the purpose of the training [21] and therefore what is being assessed as an outcome. The inclusion of clear learning outcomes as well as assessment in dementia education and training should be considered; accreditation for dementia specialist staff may incentivise this practice.

The audit highlighted that training was performing well in terms of the methods of delivery; the majority of the packages used face to face approaches to teaching. This is in line with previous research in the field suggesting this method achieves high impact [16], with the caveat that impactful face to face training should also provide opportunities for interaction. Fewer of the reported packages used e-learning, a positive finding given the literature indicates that this is a less effective form of teaching [16] and the recent National Audit of Dementia conducted in hospitals by the Royal College of Psychiatrists in England and Wales found that staff who had only received e learning training felt the least prepared to care for people with dementia [22].

The current audit found that there was a good coverage of education and training provided at Tier 1; the topic if ‘*dementia awareness’* was addressed by many of the packages and corresponded well to the LOs described in The Framework. This is likely to reflect the commitment from DoH in England that all health and social care staff should be dementia aware by 2015 [7]. However, ancillary staff such as porters and staff provided by external companies may still be overlooked, the current findings reported that few packages were targeted to these staff groups. This corresponds with findings from the 2019 National Audit of Dementia [22] which identified that the requirement for staff to have dementia training was detailed in fewer than 40% of contracts with external providers.

In terms of the content of the training provided in the packages reported in the current audit, whilst Tier 1 was covered comprehensively, there was greater variation in the degree to which Tier 2 and tier 3 learning outcomes were met. Overall, fewer than 40% of learning outcomes overall were addressed in the packages reported. The subjects ‘*communication, interaction & behaviour’* and ‘*person-centred care’* were addressed thoroughly in terms of the learning outcomes that were covered by training packages, and were also the subjects most frequently targeted in training packages. As a finding this was unsurprising given the policy driven approach to provision of person-centred care that has grown since the inception of the concept of personhood in the field [23]. Additionally, person-centred care and communication are pertinent to all professional roles and services, whereas other subject areas may be considered specialist or role specific. Countries such as Ireland [24] and the Netherlands [25] have also adopted person-centred stances on dementia care in the past 10 years, embedding this into their national dementia strategies. In countries that have adopted similar approaches to person centred care, we may anticipate similar issues with education and training may arise i.e. a greater degree of generalised training in person centred care rather than specialist role specific training.

Only 23% of the LOs for ‘r*esearch & evidence based practice’* were addressed, poorer coverage may reflect a lack of understanding regarding contemporary issues and practice. Given that current UK based policy from the National Institute for Health and Care Excellence requires everyone who is diagnosed with dementia to be offered research participation and emphasises evidence-based services, new or updated training in this domain is recommended. The subjects ‘*pharmacological interventions in dementia care’* and ‘*end of life dementia care’* were less likely to be the targeted by training. These subjects may have a more limited audience; specialist rather than generic dementia roles. In and of itself this is not problematic, but there were also fewer LOs covered in these subjects suggesting that the training currently offered does not reflect that recommended standards identified in the Framework. This is particularly important given controversial issues that can arise in fields such as prescribing, for example the overuse of antipsychotics in dementia care [26]. Fewer LOs covered in the subject ‘*equality diversity & inclusion’* suggest the same issue. Training in these subject areas may benefit from being further developed or updated.

The reach of the packages was interrogated by investigating how many learners had undertaken the training and the number of times the training had been delivered. Although prima facie it is good to see training reaching large numbers of learners, where this occurred in many cases the training had been delivered multiple times. Training that is delivered multiple times may also be predisposed to be shorter and this suggestion is perhaps corroborated by the finding that most of the packages did not exceed the eight-hour duration found in our related literature review to be the threshold for training to impact knowledge and behaviour [16]. In developing new and updating existing training packages these issues should be considered; as it is important not to prioritise accessibility over quality and impact.

At the time of this audit the original Framework had only just been released and therefore training providers could not have planned their training to deliver against its learning outcomes. This may explain the large number who started but did not complete the survey. A further limitation is that we cannot be certain whether the responses reflect the full extent of dementia training and education in England as it is possible that many organisations who deliver training did not complete the survey. However, we made every attempt to bring this to relevant audiences through a variety of media to give all an opportunity to respond, and we were able to analyse reports on 386 training packages, which is a substantial number. Future research may consider repeating the audit to consider whether education and training now reflects The Framework to a greater degree.

## Conclusion

As argued by Surr et al. [16], adapted from Opfer & Pedder [27], the health and social care workforce education is a complex system, with many facets at individual, meso, and macro (institutional) levels that must be understood to provide effective conditions for learning. However, in reviewing the current education and training offered in the UK in relation to best practice evidence, it is apparent that immediate changes to the content of training can be implemented that will better equip the dementia workforce to deliver high quality person-centred care. In particular although dementia awareness training is covered effectively, fewer than 40% of The Dementia Training Standards Framework learning outcomes targeted to staff with regular contact with people with dementia or in leadership roles were covered by the reported packages. Training targeting the subjects ‘*pharmacological interventions in dementia care’*, ‘*leadership’* and ‘e*nd of life care’* should be enhanced. Although many of the reported packages used delivery methods considered to be effective, on the while the training was not of sufficient duration to derive impact.

In the UK, findings from this audit have been used to inform quality improvement initiatives, such as the guidance document *Managing Success* which accompanies The Dementia Training Standards Framework [28]. These findings are also relevant to countries outside of the UK in the short and long term who may be seeking to review or develop their national training.

## Supplementary information

**Additional file 1:** Sample of Audit.

## Data Availability

The datasets used and/or analysed during the current study are available from the corresponding author on reasonable request.

## Abbreviations

CPD: Continuing Professional Development.
DoH: Department of Health
HEE: Health Education England (HEE)
LO: Learning Outcome
NHS: National Health Service
NICE: National Institute for Health and Care Excellence
NVQ: National Vocational Qualification
R&D: Research and Development (R&D)
UK: United Kingdom

## Footnotes

1 Routing was employed in the online version based on participants’ responses; respondents would not have been shown all questions. Questions regarding coverage of subjects and learning outcomes were repeated for each training package provided.
