# Supplementary material for: An audit of dementia education and training in UK health and social care: a comparison with national benchmark standards
Source: BMC Health Serv Res. 2019 Oct 21;19:711. doi: 10.1186/s12913-019-4510-6 (PMC6802111; doi:10.1186/s12913-019-4510-6)
Supplement: Supplementary file 1 — Additional file 1: Sample of Audit. [file 12913_2019_4510_MOESM1_ESM.pdf]

What Works? In dementia training and education

National Audit of Dementia Training and Education - Survey 1

The aim of this questionnaire is to capture information about training and education on dementia for health and social care staff; whether this is at a basic or sophisticated level, and inclusive of dementia-specific components of broader care-related programmes. We are keen to hear from training/education providers, commissioners, and those who buy-in training/education for their staff or send their staff to external courses. This could include face-to-face learning, online based courses, mentoring schemes or educational materials.

This survey may take between 10 minutes and an hour to complete, dependent on your organisation's involvement in training/education provisions and the quantity and complexities of packages available. **You will not be able to save your progress to return to at a later date**, so it is important that you allow yourself enough time to complete the survey. However, should you have a number of programmes to tell us about, it is possible to complete the survey more than once if necessary.

It is recommended that you have some basic information about your training package(s) to hand before starting the survey if applicable, as this will save time. If you are unsure about any elements of the training you provide, we are happy to receive responses from more than one member of your organisation should they have better knowledge of different packages/modules. Please share this link with your colleagues.

The survey will request the following information:

- **Detailed content of training/education packages**
- **Learning outcomes**
- **Development of the training (if relevant)**
- **Attendance/basic demographic information of trainees**
- **Frequency of training delivery**
- **Costing of training and materials**
- **Evaluations where applicable**

Please read the information sheet on the next page carefully prior to completing this questionnaire.

Information Sheet

The What Works Study

We are carrying out research commissioned by the Department of Health, on behalf of Health Education England and supported by Skills for Care and Skills for Health. We have been asked to find out what ingredients lead to effective dementia training and education that enhances staff knowledge, skills and positive attitudes towards providing good dementia care. In order to do this we need to find out what training on dementia is currently being delivered/commissioned around the UK.

What do I have to do?

We are asking all care providers, training providers and training commissioners, to complete an online national survey about the training they deliver or implement in their organisation. The survey aims to collate information with regards to the nature of current training and education packages and where they have been delivered. The survey should take no longer than 20 minutes.

What will be done with the information collected?

We will use the information to develop a picture of dementia training and education in the UK. A second survey has been developed to find out more about staff knowledge and skills. Where you have indicated that training has been delivered, we may ask you to help us access staff who have completed a specific training package, whom we will invite to take part in the second survey of those who have received the training. We will also be selecting some sites where training seems to have been particularly effective, to take part in an in-depth case study later in the research. This would be voluntary so by completing this survey you are not committing to taking part in any future elements of the research. We will write a report about what we find out through this survey but your organisation’s contribution will be kept anonymous. We’ll send you a summary of what we find.

Will the information be kept confidential?

Yes, all information collected through this survey will be kept anonymous and confidential. Only the research team will have access to the data. As part of an agreement with a team of researchers within the Faculty of Health and Medicine at Lancaster University, some survey responses may be made available to them for use in concurrent research. This will allow us to share the resources required in obtaining similar data, and reduce the survey burden on organisations. You have the opportunity to opt out of this at the bottom of this page. Any data and information we collect for the study will be in full compliance with the Data Protection Act (1998).

Do I have to take part?

You do not have to complete the survey if you do not wish to participate in the study, and you do not have to provide a reason. You can also withdraw from the study up until end of November 2015.

What are the risks and benefits of taking part?

The risks related to completing a survey are minimal. However participating in the study has a number of benefits. By completing the survey about your training, you are ensuring that your training package is considered as a potential package to be followed up later in the study to assess how effective it is. Sites that are approached to take part in the later case studies will have an opportunity to be named in relation to good practice (providing that we are given explicit permission to do so; otherwise all findings are completely confidential and reported anonymously). However, we can only conduct further study into those training providers/packages that are included in this survey.

Ethics

All research is looked at by an independent group of people, called a research ethics committee to protect your interests. This research project was given a favourable opinion by Leeds Beckett University Research Ethics committee on 30th July 2015.

If you have any further questions please contact the research team.
E-mail: whatworks@leedsbeckett.ac.uk

This is independent research commissioned and funded by the Department of Health Policy Research Programme (Understanding Effective Dementia Workforce Education and Training: A Formative Evaluation (DeWET Evaluation), PR-R10-0514-12006).

Q1.1 **Please tick this box to confirm that you have read and understood the participation information sheet and agree to participate in this survey (you will not be able to continue unless this box is ticked).**

- ❑ I understand the information provided and agree to participate in this survey

Q1.2 **If you do not wish for your response data to be shared with relevant members of the Faculty of Health and Medicine at Lancaster University, please tick this box.**

- ❑ I do not wish for my responses to be shared with Lancaster University

Q1.3 Which of the following best describes your organisation?

- ❑ Training provider (e.g. Private company, Higher Education Institution)
- ❑ Care provider (e.g. Acute NHS Trust, Community Pharmacy)
- ❑ Commissioning Group or Clinical Network (e.g. LET B, Local Authority, CCG, SCN)

Q1.4 Training provider - please specify type

- ❑ University
- ❑ Private/contract training company
- ❑ Charitable organisation
- ❑ Other

Please specify: ____

Q1.5 Care provider - please specify type

- ❑ Primary care
- ❑ Acute care
- ❑ Charitable care organisation
- ❑ Home care/domicillary care
- ❑ Community or Mental health Trust
- ❑ Residential or nursing home
- ❑ Other

Please specify: ____

Q1.6 Training provider - has your organisation's training in dementia been delivered to health and social care staff?

- ❑ Yes
- ❑ No
- ❑ Unsure

Q1.7 Care provider - does your organisation do any of the following (please tick all that apply):

- ❑ Provide staff training or education in dementia (developed in-house)
- ❑ Buy-in staff training or education in dementia from another organisation
- ❑ Send staff to externally provided training or education programmes in dementia
- ❑ No - none of the above
- ❑ Unsure

Q1.8 Commissioners and Clinical networks - are you involved in the provision of dementia training/education in any way, for health and social care staff?

- ❑ Yes
- ❑ No
- ❑ Unsure

Q1.9 Training providers - How many dementia related training courses/programmes do you wish to tell us about? (If you have more than 6 you may need to complete this survey twice)

- ❑ 1
- ❑ 2
- ❑ 3
- ❑ 4
- ❑ 5
- ❑ 6

Q1.10 Care providers - How many dementia related training courses/programmes do you wish to tell us about? (If you have more than 6 you may need to complete this survey twice)

- ❑ 1
- ❑ 2
- ❑ 3
- ❑ 4
- ❑ 5
- ❑ 6

Q1.11 Commissioners and Clinical Networks - How many dementia related training courses/programmes do you wish to tell us about? (If you have more than 6 you may need to complete this survey twice)

- ❑ 1
- ❑ 2
- ❑ 3
- ❑ 4
- ❑ 5
- ❑ 6

Section 2 - For Care Organisations

About you (this will be used to contact you if we wish to undertake further research on the outcomes of the training you tell us about):

Q2.1 Your name:

____

Q2.2 The organisation you are completing this survey on the behalf of:

____

Q2.3 Your email address:

____

Q2.4 Your telephone number:

____

Section 2: For Training Providers

About you (this will be used to contact you if we wish to undertake further research on the outcomes of the training you tell us about):

Q2.22 Your name:

____

Q2.23 The organisation you are completing this survey on the behalf of:

____

Q2.24 Your email address:

____

Q2.25 Your telephone number:

____

Section 2: For Commissioners and Clinical networks

About you (this will be used to contact you if we wish to undertake further research on the outcomes of the training you tell us about):

Q2.52 Your name:

____

Q2.53 The organisation you are completing this survey on the behalf of:

____

Q2.54 Your email address:

____

Q2.55 Your telephone number:

____

Training course/package 1:

Q2.56 Name of training package:

____

Q2.57 What issues did you consider when choosing to purchase/develop this particular training? (Please tick all that apply)

- ❑ Content met identified training need
- ❑ Provided locally
- ❑ Affordable cost
- ❑ Reputation of provider
- ❑ Delivery method met our needs
- ❑ Training could be delivered in a timely manner
- ❑ Sustainable delivery
- ❑ Other

Please specify:

____

Q2.58 How many times has this training package/programme been delivered? (Please leave blank if unknown)

____

Q2.59 Which type of organisations has this training been provided to? (Please tick all that apply)

- ❑ Acute care
- ❑ Primary care
- ❑ Mental health trusts
- ❑ Community outreach services
- ❑ Residential/nursing homes
- ❑ Community pharmacies
- ❑ Domicillary care
- ❑ Other

Please specify:

____

Q2.60 How many organisations has the training been provided to? (Please leave blank if unknown)

____

Q2.61 Approximately how many participants have received the training since January 2012? (Please leave blank if unknown)

____

Q2.62 How much does the training cost for the organisation? (If free write '0', please leave blank if unknown)

____

How much does the following cost per training cycle? (If unsure or not applicable, please leave blank)

Q2.63 Staff course fees

____

Q2.64 Release of staff

____

Q2.65 Room hire

____

Q2.66 Equipment

____

Q2.67 Release of trainer

____

Q2.68 If training was developed in-house, how much did it cost to develop?

____

Q2.69 Approximately how many staff hours went into its development?

____

Q2.70 What grade or type of staff was the training aimed towards? (Please tick all that apply)

- ❑ All
- ❑ Ancillary and clerical (e.g. porter, secretary, domestic staff)
- ❑ Unqualified clinical/care staff (e.g. nursing assistant, support worker, residential care worker)
- ❑ Qualified clinical/care staff (e.g. general practitioner, psychologist, nurse)
- ❑ Unit/ward/facility managers
- ❑ Service managers
- ❑ Other

Please specify:

____

Q2.71 Any additional costs for resources? e.g. Equipment, video or web-development?

____

Section 3: Level of training and education

Q3.1 Which of the following best describes the level of the training/education provided by this package/programme (as defined by the Dementia Core Skills and Knowledge Framework?)

- ❑ Awareness raising - dementia awareness raising, in terms of knowledge, skills and attitudes for all those working in health and social care
- ❑ In-depth training - knowledge, skills and attitudes for roles involving regular contact with people living with dementia
- ❑ Expert leaders training - enhancingthe knowledge, skills and attitudes for key staff (experts) working with people living with dementia designed to support them to play leadership roles

Q3.2 Which of the following are covered by the programme? (Please tick all that apply)

	Yes	Unsure	No
Dementia awareness	❑	❑	❑
Dementia identification, assessment and diagnosis (Diagnosing dementia)	❑	❑	❑
Dementia risk reduction and prevention	❑	❑	❑
Person-centred dementia care (understanding and responding to the person with dementia as an individual)	❑	❑	❑
Communication, interaction and behaviour in dementia care (effectively communicating with people with dementia)	❑	❑	❑
Health and well-being in dementia care (Helping people with dementia maintain their physical and psychological health)	❑	❑	❑
Pharmacological interventions in dementia care (prescription of medicines)	❑	❑	❑
Living well with dementia and promoting independence	❑	❑	❑
Families and carers as partners in dementia care	❑	❑	❑
Equality diversity and inclusion in dementia care	❑	❑	❑

Law, ethics and safeguarding in dementia care (Duty of care)	❑	❑	❑
End of life dementia care	❑	❑	❑
Research and evidence-based practice in dementia care	❑	❑	❑
Leadership in transforming dementia care	❑	❑	❑

Q3.3 **Awareness training** - Does your training address any of the following learning outcomes?

	Yes	Unsure	No
**The learner will...** know what is meant by the term 'dementia'	❑	❑	❑
be aware of the prevalence of dementia in the UK population	❑	❑	❑
be able to recognise signs of dementia and also be aware that these signs may be associated with other conditions or circumstances	❑	❑	❑
know what actions individuals can take to reduce their risk of dementia, or to delay onset	❑	❑	❑
know why early diagnosis of dementia is important	❑	❑	❑
know the actions that people affected by dementia can take in order to live as well as possible after diagnosis	❑	❑	❑
understand the importance of recognising the person with dementia as a unique individual	❑	❑	❑
be aware of the impact of dementia on individuals, families and society	❑	❑	❑
be able to communicate effectively and compassionately with individuals who have dementia	❑	❑	❑
understand reasons why a person with dementia may exhibit signs of distress and how behaviours seen in people with dementia may be a means for communicating unmet needs	❑	❑	❑

	Yes	Unsure	No
be able to signpost individuals, families and carers to dementia advice, support and information	❑	❑	❑

Q3.4 **Dementia Identification, Assessment and Diagnosis** - Does your training address any of the following learning outcomes?

	Yes	Unsure	No
**The learner will...** know the most common types of dementia in the UK and their underlying causes	❑	❑	❑
understand the signs and symptoms of dementia that would indicate the need for further assessment	❑	❑	❑
know why early diagnosis of dementia is important and the likely outcomes if assessment and treatment is delayed	❑	❑	❑
know the progressive nature of dementia and some of the major impairments and difficulties people may face as dementia progresses	❑	❑	❑
understand the criteria and the process to be used to gain a diagnosis	❑	❑	❑
be able to explain the need for an investigation of signs of dementia with sensitivity and in a way that is appropriate to the person	❑	❑	❑
be able to appropriately refer patients to access specialist services and support networks	❑	❑	❑
understand the different types of dementia, the stages or variants of these diseases and their primary symptoms	❑	❑	❑
understand how to differentiate between dementia, delirium, depression and other conditions presenting with similar symptoms	❑	❑	❑
be able to undertake a comprehensive assessment for dementia utilising appropriate investigations and tools	❑	❑	❑
be able to establish a differential diagnosis of dementia and the underlying disease processes, where appropriate to role	❑	❑	❑
be aware of the potential impact of diagnostic errors	❑	❑	❑

be able to act on the findings in partnership with people affected by dementia and the multi-professional team	❑	❑	❑
be aware of the experience of a person with dementia and their family and carers and be able to communicate with sensitivity about the diagnosis of dementia and related implications	❑	❑	❑
know how to enrol the person with dementia in post-diagnosis support services and advanced care planning	❑	❑	❑
understand the particular impact of a diagnosis for younger people with dementia and their families	❑	❑	❑
understand the needs of people with learning disabilities and dementia	❑	❑	❑
understand the importance of equal access to dementia assessment and diagnosis for people from diverse communities	❑	❑	❑
be able to document assessment and diagnosis decisions	❑	❑	❑

Q3.5 **Dementia Risk Reduction and Prevention -** Does your training address any of the following learning outcomes?

	**Yes**	**Unsure**	**No**
**The learner will...** know the lifestyle factors that may increase the risk of developing certain types of dementia and how lifestyle changes may delay the onset and severity of certain types of dementia	❑	❑	❑
understand motivational factors that may impact on the ability to make changes	❑	❑	❑
be aware of the challenges to healthy living that may be experienced by different socio-economic and/or ethnic groups	❑	❑	❑
be able to signpost sources of health promotion information and support	❑	❑	❑

know how to effectively communicate messages about healthy living according to the abilities and needs of individuals	❑	❑	❑
be aware of dementia risk reduction evidence-based research and national health promotion strategies	❑	❑	❑
be able to develop and disseminate health promotion information and advice	❑	❑	❑
be able to encourage behavioural change in individuals and organisations to promote health and well-being, reduce risk and potentially delay the onset and severity of certain types of dementia	❑	❑	❑
understand the importance of an approach to risk reduction which challenges myths and stigma	❑	❑	❑
be able to monitor, evaluate and improve the effectiveness of health promotion activities	❑	❑	❑

Q3.6 **Person Centred Dementia Care** - Does your training address any of the following learning outcomes?

	Yes	Unsure	No
**The learner will...** understand the principles of person-centred dementia care: i.e. the human value of people with dementia, regardless of age or cognitive impairment, and those who care for them; the individuality of people with dementia, with their unique personality and life experiences among the influences on their response to the dementia; the importance of the perspective of the person with dementia; the importance of relationships and interactions with others to the person with dementia, and their potential for promoting well-being	❑	❑	❑
understand how person-centred care can provide insights into the experiences of the person with dementia and support care approaches and solutions to meet individual needs	❑	❑	❑
understand the role of family and carers in person-centred care and support of people with dementia	❑	❑	❑

understand how a person-centred approach can be implemented, including the use of advance planning and life story work	❑	❑	❑
understand that a person’s needs may change as the disease progresses	❑	❑	❑
know how to adapt the physical environment to meet the changing needs of people with dementia	❑	❑	❑
understand the significance of a person’s background, culture and experiences when providing their care	❑	❑	❑
understand the importance of clear documentation to communicate the care needs of the person with dementia	❑	❑	❑
understand the value of person-centred care in therapeutic relationships and communication	❑	❑	❑
understand the importance of person-centred approaches in the management and development of services	❑	❑	❑
be able to incorporate person-centred approaches in the management and development of services	❑	❑	❑

Q3.7 **Communication, Interaction and Behaviour in Dementia Care** - Does your training address any of the following learning outcomes?

	Yes	Unsure	No
**The learner will...** understand the importance of effective communication in dementia care	❑	❑	❑
understand the impact of memory and language difficulties on communication	❑	❑	❑
be able to demonstrate active listening skills	❑	❑	❑
be able to gain a person’s attention before asking a question or beginning a task with them	❑	❑	❑
understand the importance of speaking clearly, calmly and with patience	❑	❑	❑

know how to adapt the environment to minimise sensory difficulties experienced by an individual with dementia	❑	❑	❑
know the importance of ensuring that individuals have any required support (e.g. spectacles, hearing aids) to enable successful communication	❑	❑	❑
know how life story information may enable or support more effective communication	❑	❑	❑
understand the importance of effective communication with family and carers and the expertise that they may be able to offer to support effective communication with the person with dementia	❑	❑	❑
be able to adapt communication techniques according to the different abilities and preferences of people with dementia	❑	❑	❑
be aware of the importance of non-verbal communication e.g. body language, visual images and the appropriate use of touch	❑	❑	❑
understand that the behaviour of a person with dementia is a form of communication and how behaviours seen in people with dementia may be a means for communicating unmet needs	❑	❑	❑
understand how a person's feelings and perception may affect their behaviour	❑	❑	❑
understand how the behaviour of others might affect a person with dementia	❑	❑	❑
understand common causes of distressed behaviour by people with dementia	❑	❑	❑
be able to recognise distressed behaviour and provide a range of responses to comfort or reassure the person with dementia	❑	❑	❑

	Yes	Unsure	No
be able to contribute to the development of practices and services that meet the communication needs of people with dementia	❑	❑	❑
be able to promote effective communication in a health and care environment	❑	❑	❑

Q3.8 **Health and Wellbeing in Dementia Care** - Does your training address any of the following learning outcomes?

	Yes	Unsure	No
**The learner will...** understand the importance for individuals with dementia to maintain good physical and mental health through nutrition, exercise and a healthy life style that includes social engagement	❑	❑	❑
be aware of anticipating an individual's health needs e.g. to prevent fatigue, falls, dehydration and hunger	❑	❑	❑
know the action to take in response to identification of fatigue, dehydration and hunger	❑	❑	❑
know how to recognise and manage pain in people with dementia	❑	❑	❑
be able to support an individual in maintaining personal appearance and hygiene	❑	❑	❑
be aware of the impact of delirium, depression and social stressors	❑	❑	❑
understand triggers and responses to stressed or distressed behaviours	❑	❑	❑
understand the role of family and carers in supporting the health and well-being of people with dementia	❑	❑	❑
be aware of the benefits and limitations of medication to manage behavioural and psychological issues including associated risks	❑	❑	❑
be able to support individuals in undertaking psycho-social interventions including validation, counselling, reminiscence and life story work	❑	❑	❑
be aware of the role of therapeutic work including complementary therapies and sensory stimulation	❑	❑	❑

know how to support people with dementia to access local services and referral pathways including voluntary and community services which would promote their physical and mental health.	❑	❑	❑
understand the signs of dementia, depression and delirium and appropriate responses and treatment options	❑	❑	❑
understand the complexity of ageing and co-morbidity in dementia	❑	❑	❑
understand the benefits and implications of pharmacological interventions that may enhance memory	❑	❑	❑
understand the evidence for the effectiveness of different psycho-social approaches in different situations	❑	❑	❑
understand the principles and key aspects of psycho-social approaches used to enhance the well-being of people with dementia	❑	❑	❑
be aware of new and emerging knowledge of psycho-social approaches that can be used to enhance the well-being of people with dementia	❑	❑	❑

Q3.9 **Pharmacological Interventions in Dementia Care** - Does your training address any of the following learning outcomes?

	**Yes**	**Unsure**	**No**
**The learner will...** know the most common medications used to treat the symptoms of dementia	❑	❑	❑
know the main risks and benefits of using anti-psychotics, anti-depressants, anxiolytics, anticonvulsants and cognitive enhancers	❑	❑	❑
be aware of issues around polypharmacy for people with dementia	❑	❑	❑
understand the importance of recording and reporting side effects and/or adverse reactions to medication	❑	❑	❑

be able to administer and review medication safely and appropriately in consultation with people affected by dementia	❑	❑	❑
understand the range of cognitive enhancers, what they do, criteria for eligibility and sources of guidance	❑	❑	❑
understand processes for assessing and prescribing cognitive enhancers	❑	❑	❑
understand the range of drugs to manage behavioural and psychological symptoms of dementia and when such drugs should or should not be used	❑	❑	❑
understand the ethical issues around drug treatments in the care of people living with dementia	❑	❑	❑
understand contra-indications for prescribing anxiolytics and anti-psychotic medication	❑	❑	❑
understand the range of medication to address common physical health problems of people with dementia, including the risks associated with how these drugs may interact with cognitive enhancers and drugs prescribed to address behavioural and psychological issues	❑	❑	❑
understand the importance of regular reviews of prescribed medication	❑	❑	❑
understand how to assess pain experienced by people with dementia and prescribing practice to address pain effectively	❑	❑	❑
be aware of new and emerging knowledge of pharmacological interventions that can be used to enhance the well-being of people with dementia	❑	❑	❑

Q3.10 **Living Well with Dementia and Promoting Independence** - Does your training address any of the following learning outcomes?

	**Yes**	**Unsure**	**No**
**The learner will...** understand the importance of physical activity (including access to outside space) in maintaining a person's independence and abilities	❑	❑	❑

be able to support individuals to meet their daily living needs	❑	❑	❑
be able to support individuals to continue their interests, social life and community involvement and know why this is important	❑	❑	❑
know about community initiatives such as the development of dementia friendly environments	❑	❑	❑
understand the needs of individuals for day to day closeness with others e.g. sharing thoughts and feelings	❑	❑	❑
understand how to recognise and respond to cultural, spiritual and sexual needs of people with dementia	❑	❑	❑
understand the role of family and carers in enabling people with dementia to live well	❑	❑	❑
understand how activities can be adapted to suit an individual's changing needs	❑	❑	❑
be able to incorporate assistive technology to support self-care and meaningful activity	❑	❑	❑
be able to develop strategies to reduce the struggle with unfamiliar environments	❑	❑	❑
be aware of ways to adapt the physical environment to promote independence, privacy, orientation and safety (e.g. to reduce risk of falls)	❑	❑	❑
know about perceptual distortions that may occur in dementia and how the impact of such distortion can be minimised by changes to the environment	❑	❑	❑
be able to contribute to the development of practices and services that meet the individual needs of people with dementia	❑	❑	❑
understand the principles, processes and options for self-directed support	❑	❑	❑

	Yes	Unsure	No
be able to support the person with dementia and their family to access self-directed support if desired	❑	❑	❑
be able to provide dementia specific advice and guidance on adapting the physical and social environment to ensure physical safety and emotional security	❑	❑	❑
be able to lead on the introduction of assistive technology to support self-care and meaningful activity	❑	❑	❑

Q3.11 **Families and Carers as Partners in Dementia Care** - Does your training address any of the following learning outcomes?

	Yes	Unsure	No
**The learner will...** understand the significance of family, carers and social networks in planning and providing care	❑	❑	❑
understand the importance of developing partnerships with family members and carers	❑	❑	❑
understand the impact that caring for a person with dementia in the family may have on relationships	❑	❑	❑
understand the importance of recognising and assessing a carer's own needs, including respite	❑	❑	❑
be aware of the complexity and diversity in family arrangements	❑	❑	❑
be aware that the needs of carers and the person with dementia may not always be the same	❑	❑	❑
understand potential socio-cultural differences in the perception of the care giving role	❑	❑	❑
be aware of the impact on younger carers and their concerns	❑	❑	❑
be able to communicate compassionately, effectively and in a timely manner with care partners	❑	❑	❑
be able to support family carers to access and use information and local support networks	❑	❑	❑

be able to support family carers in considering options and making decisions	❑	❑	❑
be able to gather information about a person's history and preferences from family carers	❑	❑	❑
be able to contribute to the development of practices and services that meet the needs of families and carers	❑	❑	❑
understand methods to assess a carer's psychological and practical needs and the relevant support available	❑	❑	❑
understand the potential for dilemmas arising where there are differing needs between people with dementia and their carers	❑	❑	❑
understand the role of personalisation in care e.g. the impact of access to personal budgets	❑	❑	❑
understand legislation relevant to carers and carers rights	❑	❑	❑
be able to signpost carers for further support around legal issues (e.g. lasting power of attorney)	❑	❑	❑

Q3.12 **Equality, Diversity and Inclusion in Dementia Care** - Does your training address any of the following learning outcomes?

	Yes	Unsure	No
**The learner will...** be aware of cultural diversity and equality issues, and how they may impact on people with dementia	❑	❑	❑
be able to adapt assessment and care planning taking account of equality issues (e.g. cultural diversity, disabilities, gender and sexual orientation)	❑	❑	❑
understand diversity in family arrangements and the local community	❑	❑	❑
be aware of the stigma, myths and stereotypes associated with dementia	❑	❑	❑
be aware of the prevalence and impact of younger onset dementia	❑	❑	❑

	Yes	Unsure	No
be aware of legislation to support carers, including young carers	❑	❑	❑
understand the additional concerns of younger carers	❑	❑	❑
be aware of the impact of dementia on people with learning disabilities	❑	❑	❑
be aware of socio-cultural differences in the perception of the care giving role e.g. based on gender	❑	❑	❑
be able to actively challenge any discriminatory practice that may compromise a person's right to dignity, respect and safety	❑	❑	❑
understand the impact that discrimination and stigma may have on the life of the person with dementia, their family and carers	❑	❑	❑
be able to lead practice and an organisational culture that values and respects the diversity of individuals	❑	❑	❑
understand legislation relevant to equality, diversity and human rights	❑	❑	❑

Q3.13 **Law, Ethics and Safeguarding in Dementia Care** - Does your training address any of the following learning outcomes?

	**Yes**	**Unsure**	**No**
**The learner will...** understand how duty of care contributes to safe practice	❑	❑	❑
be aware of dilemmas that may arise between the duty of care and an individual's rights and carers wishes	❑	❑	❑
be able to communicate effectively about proposed treatment or care to enable people with dementia to make informed choices as far as practicable	❑	❑	❑
understand the protocols regarding consent to treatment or care for people who may lack mental capacity	❑	❑	❑
understand how 'best interests' decisions may need to be made for those lacking capacity	❑	❑	❑

	Yes	Unsure	No
know how advance directives can be used to provide information about the wishes of an individual	❑	❑	❑
be able to recognise a range of factors which may indicate neglect, abusive or exploitative practice	❑	❑	❑
know what to do if neglect, abusive or exploitative practice is suspected, including how to raise concerns within local safeguarding or whistle blowing procedures	❑	❑	❑
be aware of key legislation relevant to mental capacity, deprivation of liberty, equality and human rights	❑	❑	❑
understand the options available when informed consent may be compromised	❑	❑	❑
be able to respond to safeguarding alerts / referrals	❑	❑	❑
know the evidence based approaches and techniques to assess neglect or abuse	❑	❑	❑
understand the roles and responsibilities of the different agencies involved in investigating allegations of neglect or abuse	❑	❑	❑
understand the importance of sharing safeguarding information with the relevant agencies	❑	❑	❑
know the actions to take if there are barriers to alerting the relevant agencies	❑	❑	❑
understand key legislation relevant to mental capacity, deprivation of liberty, equality and human rights	❑	❑	❑

Q3.14 **End of Life Dementia Care -** Does your training address any of the following learning outcomes?

	Yes	Unsure	No
**The learner will...** understand the use of end of life care pathways and individualised care plans taking into account psycho-social needs	❑	❑	❑
understand how advanced decisions/directives and best interest decision will affect caring activities	❑	❑	❑

	Yes	Unsure	No
know how to recognise and manage pain in people with advanced dementia	❑	❑	❑
be able to identify symptoms associated with end of life and how these symptoms can be managed with care and compassion	❑	❑	❑
be aware of concerns and needs affecting younger people at the end of life	❑	❑	❑
be aware of the needs of bereaved families and friends including the potential for conflicting emotions	❑	❑	❑
be able to support family and friends to celebrate the life of the deceased person	❑	❑	❑
be aware of cultural and religious differences associated with death, care of the dying and the deceased person.	❑	❑	❑
be able to contribute to the development of practices and services that meet the end of life needs of people with dementia	❑	❑	❑
understand the processes involved in deciding when a person with dementia is deemed to be at end of life	❑	❑	❑
be able to provide dementia specific advice and guidance on end of life care	❑	❑	❑

Q3.15 **Research and Evidence Based Practice in Dementia Care** - Does your training address any of the following learning outcomes?

	Yes	Unsure	No
**The learner will...** understand the difference between service evaluation and research	❑	❑	❑
be able to participate in service evaluation and research in the workplace	❑	❑	❑
understand how people affected by dementia may be involved in service evaluation and research	❑	❑	❑
understand systematic research methods to facilitate evidence-based practice	❑	❑	❑

	Yes	Unsure	No
understand the range of evidence that informs decision-making, care practice and service delivery	❑	❑	❑
understand approaches to evaluating services and measuring impact, including the use of outcomes reported by people affected by dementia	❑	❑	❑
understand the ethical issues related to conducting research with people who have a cognitive impairment	❑	❑	❑
be able to disseminate research findings clearly and accurately in written reports or verbal presentations	❑	❑	❑
understand the importance of continuing professional development to ensure the methods used are robust, valid and reliable	❑	❑	❑

Q3.16 **Leadership in transforming Dementia Care** - Does your training address any of the following learning outcomes?

	**Yes**	**Unsure**	**No**
**The learner will...** understand the key drivers and policies which influence national dementia strategy and service development	❑	❑	❑
be aware of evidence-based research, innovations and developments in dementia interventions and care	❑	❑	❑
be able to disseminate and promote new and evidence-based practice and to challenge poor practice	❑	❑	❑
be able to plan care to promote the use of appropriate, specific, evidence based interventions	❑	❑	❑
understand the importance of demonstrating leadership in delivering compassionate person-centred care	❑	❑	❑
understand the importance of quality assurance and service improvement	❑	❑	❑
know how to ensure team members are trained and supported to meet the needs of people with dementia	❑	❑	❑
understand the roles and responsibilities of different agencies involved in dementia care	❑	❑	❑

understand the principles of equality and diversity for access to, and delivery of services. ❑ ❑ ❑

understand the importance of collaborative working in the provision of support, care and services for people with dementia, their families and carers ❑ ❑ ❑

Q3.17 Has the impact of training been evaluated?

- ❑ Yes
- ❑ No
- ❑ Unsure

Please provide details (Including who conducted the evaluation, when it was conducted and if a report is available)

____

Q3.18 If a report or other output is available from the evaluation would you be willing to share this with us?

- ❑ Yes
- ❑ No

Q3.19 Have there been any barriers to implementing/delivering training? (Please tick all that apply)

- ❑ Time
- ❑ Resources
- ❑ Staff unable to attend
- ❑ Lack of support from management
- ❑ Other barriers
- ❑ None identified

Please give more details:

____

Q3.20 What has helped with implementing/delivering the training? (Please tick all that apply)

- ❑ Designated time
- ❑ Resources
- ❑ Staff incentives
- ❑ Support from management
- ❑ Other factors have been helpful
- ❑ None identified

Please give more details:

____

NB: Sections 4-13 have been removed for brevity. The questions in these sections are duplicates of section 2 & 3 for up to 6 packages in total

You have now reached the end of the survey; if you feel that you have more training packages available which may be of interest to our study, please feel free to complete the survey again or get in touch with us using the contact details on the next page.

THANK YOU!

We would like to thank you very much for completing this survey.

If you have any questions, please do not hesitate to e-mail us:

whatworks@leedsbeckett.ac.uk

To learn more about the What Works Project, please visit http://www.leedsbeckett.ac.uk/pages/what-works/ or follow us on twitter: @whatworksstudy
